# Performance Recovery after Contamination with Nitrogen Dioxide in a PEM Fuel Cell

**DOI:** 10.3390/molecules25051115

**Published:** 2020-03-02

**Authors:** Yasna Acevedo Gomez, Göran Lindbergh, Carina Lagergren

**Affiliations:** Applied Electrochemistry, Department of Chemical Engineering, KTH Royal Institute of Technology, 10044 Stockholm, Sweden; yasna@kth.se (Y.A.G.); gnli@kth.se (G.L.)

**Keywords:** PEM fuel cell, performance, recovery, nitrogen dioxide, contamination

## Abstract

While the market for fuel cell vehicles is increasing, these vehicles will still coexist with combustion engine vehicles on the roads and will be exposed to an environment with significant amounts of contaminants that will decrease the durability of the fuel cell. To investigate different recovery methods, in this study, a PEM fuel cell was contaminated with 100 ppm of NO_2_ at the cathode side. The possibility to recover the cell performance was studied by using different airflow rates, different current densities, and by subjecting the cell to successive polarization curves. The results show that the successive polarization curves are the best choice for recovery; it took 35 min to reach full recovery of cell performance, compared to 4.5 h of recovery with pure air at 0.5 A cm^−2^ and 110 mL min^−1^. However, the performance recovery at a current density of 0.2 A cm^−2^ and air flow 275 mL min^−1^ was done in 66 min, which is also a possible alternative. Additionally, two operation techniques were suggested and compared during 7 h of operation: air recovery and air depletion. The air recovery technique was shown to be a better choice than the air depletion technique.

## 1. Introduction

As the world is heading towards clean energy sources, the proton exchange membrane (PEM) fuel cell plays an important role, being a good alternative for the transportation sector and stationary power systems. Automobile manufacturers have been releasing electric vehicles as a viable solution to decrease greenhouse gas emissions [1]. The fuel cell vehicle is becoming popular and may be the right solution to replace internal combustion engine (ICE) vehicles in the near future [2,3]. However, the durability of the fuel cell is still an issue, where one aspect is pollutants in the air that seriously affect the performance. It is well known that the air contains unwanted contaminants that come from ICE vehicles, agriculture, and industries. As the fuel cell market grows, fuel cell vehicles must coexist with ICE vehicles on the roads. The coexistence of these two types of vehicles may lead to a dramatic decrease in the fuel cell performance, thus a recovery strategy must be considered in a real traffic situation.

Among the contaminants in air, nitrogen dioxide is one that seriously affects the performance of the PEM fuel cell but has not been completely studied in the literature. In our previous study [4], severe degradation of the cell performance was shown at different concentrations of NO_2_. For all the tests, the same total dosage of NO_2_ was added, but the possibility for the cell performance to recover after contamination differed. At higher concentrations of 50, 100, and 200 ppm NO_2_, the performance could only be partially recovered. In the study, a mechanism for NO_2_ contamination was proposed based on cyclic voltammetry (CV) observation in which NO_2_ is oxidized to NO_3_^−^ at 1.05 V, then in the negative sweep reduced to NO_2_^−^ at 0.68 V, followed by a subsequent reduction of NO_2_^−^ to N_2_O and/or NH_2_OH at potentials lower than 0.5 V. The proposed mechanism was confirmed by the detection of NO as intermediate species and N_2_O by simultaneous mass spectrometry.

Other authors have shown that the contamination can be fully recovered in some cases [5,6], almost recovered in other cases [6,7,8], or not recovered [5,9], depending on the NO_2_ concentration, exposure time, and operating conditions. Misz et al. [6] and Jing et al. [8] tested the contamination with 1 ppm NO_2_ over 1 and 100 h, respectively; the shorter exposure time resulted in fully recovered performance while performance following the longer exposure time was almost recovered after cyclic voltammetry scan as a recovery process. It is seen that long-term exposure produces an unrecoverable effect. Mohtadi et al. [9] and Uribe et al. [5] contaminated the fuel cell with 5 ppm NO_2_ over 12 and 15 h, respectively. In these cases, the result from Mohtadi et al. [9] was partially recovered cell performance, while the result from Uribe et al. [5] was fully recovered performance. The difference of these two recovery processes was that Mohtadi et al. [9] operated the cell in the range of 0.68–0.7 V and Uribe et al. [5] at 0.5 V. Our previous results [4], in agreement with the results of Chen et al. [10] and Lin et al. [11], showed that, at lower potentials in the negative sweep, reduction of nitrite occurs, and thus it is removed from the Pt-catalyst. Higher concentrations were tried by Yang et al. [7] (10, 140, and 1480 ppm) and Misz et al. [6] (10 and 15 ppm), in which performance recovery was almost reached in all of the cases after approximately 1 h with NO_2_. When it comes to long-term operation, Uribe et al. [5] showed that performance following contamination of 0.4 ppm NO_2_ for around 520 h was not recovered, probably due to the low amount of catalyst they used (17 µg Pt cm^−2^) that was quickly damaged. 

St-Pierre et al. [12] simulated performance recovery after 500 h of exposure to 0.1 ppm NO_2_. Even if they used dry air conditions in their simulation, where the performance was dramatically affected, the performance was recovered and reached its initial value. This result is contradictory to the one obtained by Uribe et al. [5] and may be due to different conditions, but unfortunately the operating conditions used were not specified in Uribe’s report. 

The aim of this study was to contribute to the improvement of the durability of the fuel cell by trying different operating conditions that influence the recovery process after NO_2_ contamination. These processes included successive polarization curves and recovery at different flow rates and current densities. In real traffic situations, exposure to high amounts of NO_2_ is unavoidable, and recovery methods that can be applied online in real fuel cell vehicles are desired. Therefore, two such realistic operation techniques were suggested and compared: consecutive recovery with air and air depletion. 

## 2. Results and Discussion

### 2.1. Performance of the Contaminated MEA

**Figure 1:**
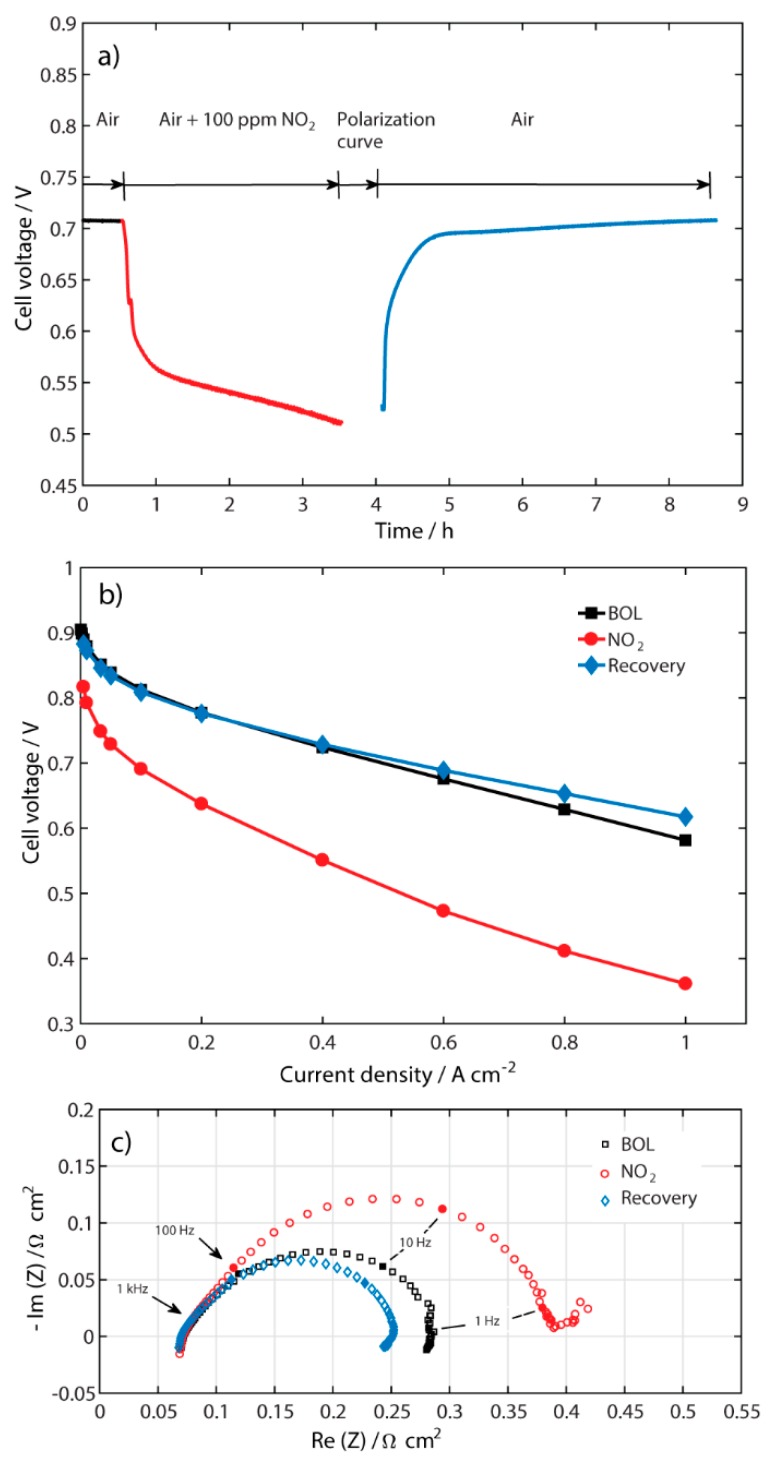
(**a**) Performance during contamination with 100 ppm NO_2_ and recovery at 0.5 A cm^−2^ and air flow 110 mL min^−1^; (**b**) polarization curves; and (**c**) galvanostatic EIS measurements at 0.5 A cm^−2^ for BOL, with NO_2_, and after recovery in cathode air flow.

The degradation of fuel cell performance upon contamination with 100 ppm of NO_2_ in air and its subsequent recovery of performance is shown in Figure 1a. The sequence of experiments was to run the cell in a galvanostatic mode at 0.5 A cm^−2^ with clean air for 30 min without contaminant, followed by the introduction of 100 ppm NO_2_ in the cathode air flow for 3 h, and then recovery of performance with pure air. Polarization curves (Figure 1b) and electrochemical impedance spectroscopy (EIS) (Figure 1c) were done at the beginning of life (BOL), after contamination with NO_2_, and after recovery with air. Figure 1a shows the dramatic performance degradation of 197 mV after 3 h of contamination. However, after switching off the NO_2_ contaminant and running the cell with clean air, the fuel cell performance was completely recovered in 4.5 h.

The polarization curves in Figure 1b show a clear contamination of NO_2_, mainly at lower current densities, where the Pt-catalyst active sites are affected by NO_2_. In this part of the curve, the contamination is related to the electrode kinetics, most likely at the cathode, which is the main contributor to the performance loss and where the contaminant is introduced. In the graph, it is also shown that the performance was completely recovered when pure air was added at 110 mL min^−1^ with a current density of 0.5 A cm^−2^. Furthermore, the recovered performance was better than at beginning of life (BOL) at high current densities, which may indicate better conductivity in the membrane due to water being produced by the ORR, while at the same time intermediate species are being reduced in the actual potential range, as mentioned in our previous publication [4].

To better diagnose the performance limitation after contamination with NO_2_ and the respective recovery in the fuel cell, EIS spectra were recorded at 0.5 A cm^−2^ and shown in Figure 1c. After contamination with NO_2_, a second semicircle is beginning to be formed at lower frequencies. However, this second semicircle disappears after the recovery process with pure air. Additionally, the polarization resistance decreases, which is in accordance with the polarization curve in Figure 1b and may be related to a better access to platinum sites after the recovery process. There is no change in the high frequency resistance (HFR), showing that the membrane resistance was not affected by contamination with NO_2_ and the recovery process.

Based on the degradation and time for performance recovery shown in Figure 1a, different air flow rates, different constant current densities, and successive polarization curves were tested during the recovery of the contaminated MEA to investigate and understand the recovery process of this contaminant.

### 2.2. Recovery at Different Air Flow Rates

**Figure 2:**
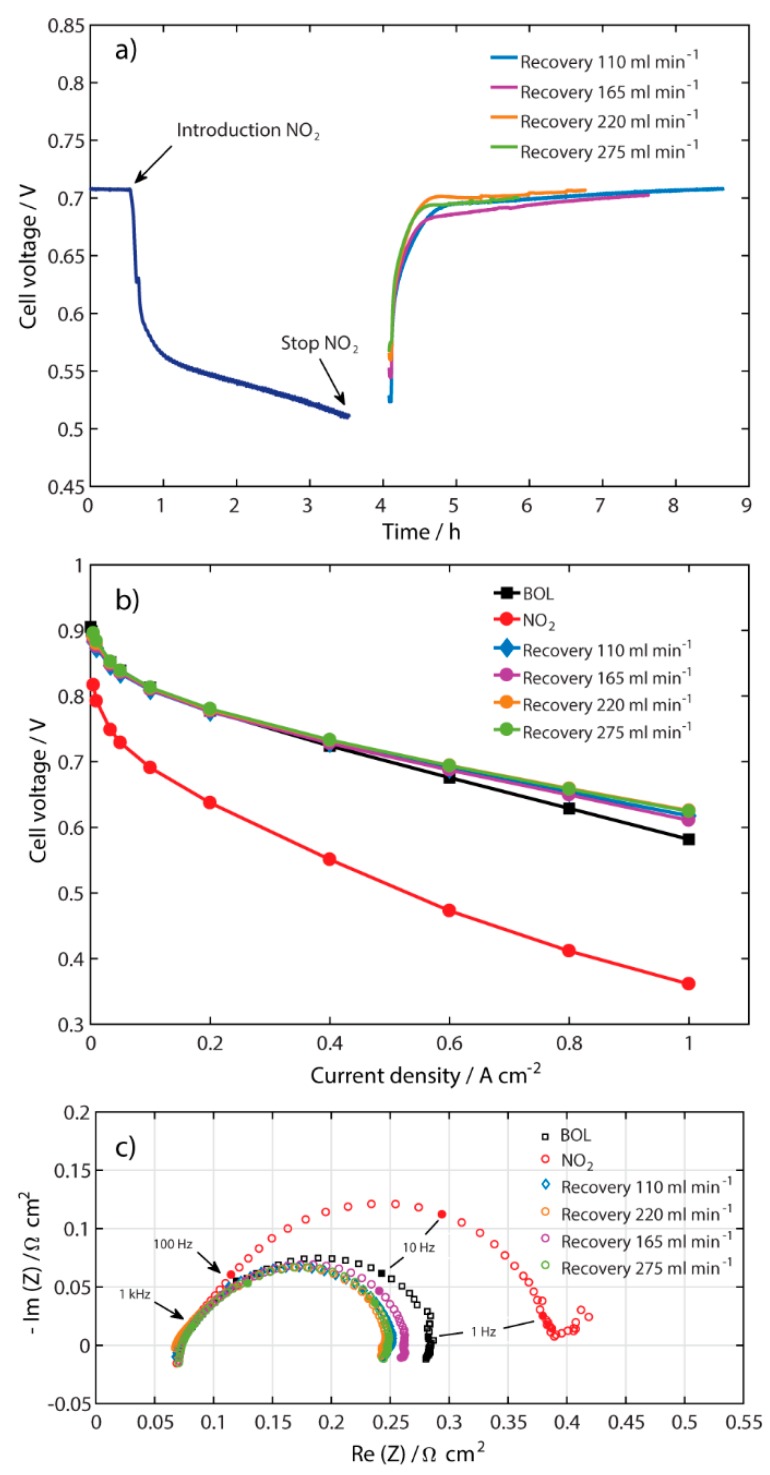
(**a**) Performance with 100 ppm NO_2_ and after recovery at different airflows 110, 165, 220, and 275 mL min^−1^ at 0.5 A cm^−2^; (**b**) polarization curves; and (**c**) galvanostatic EIS measurements at 0.5 A cm^−2^ for the same conditions as in (**a**).

To find a shorter performance recovery time for the MEA contaminated with NO_2_, different air flow rates (110, 165, 220, and 275 mL min^−1^) were tested for the recovery process at a constant current density of 0.5 A cm^−2^, as shown in Figure 2a. The time required to reach the same cell voltage as before contamination is defined as the recovery time. The same contamination sequence as described above was used and the air flow rate was changed to the desired value for the recovery of the performance. Figure 2a shows that all the curves reached their initial values after the recovery process, but after different periods of time. The faster recovery time was found to be at the highest flow rate, 275 mL min^−1^. This is a clear sign that the NO_2_ contaminant is not as well attached to the Pt-catalyst surface as sulfur compounds are [9]. As soon as clean air is introduced into the recovery process, most of the NO_2_ is removed from the Pt-catalyst. This is shown in Figure 2a by the sharp increase in cell voltage (~180 mV) within about 30 min, after which a slower relaxation period occurs until steady state is reached.

Figure 2b shows that all polarization curves overlap up to the current density of 0.4 A cm^−2^. As the current density increases further, small differences in potential can be seen between the curves at different flow rates, where the two highest air flow rates show the best performance. The performance after the recovery process is higher than at BOL for all the different air flow rates, in the same way as in Figure 1b.

The EIS spectra after the recovery process at different air flow rates are depicted in Figure 2c. It can be seen that there is no significant difference in the HFR where the spectra intercept the real axis. After the recovery process, all of the spectra show a lower polarization resistance when compared with BOL, which is in accordance with Figure 2b. It might be possible that some Pt-sites were activated after the recovery process with pure air.

### 2.3. Recovery at Different Current Densities

**Figure 3:**
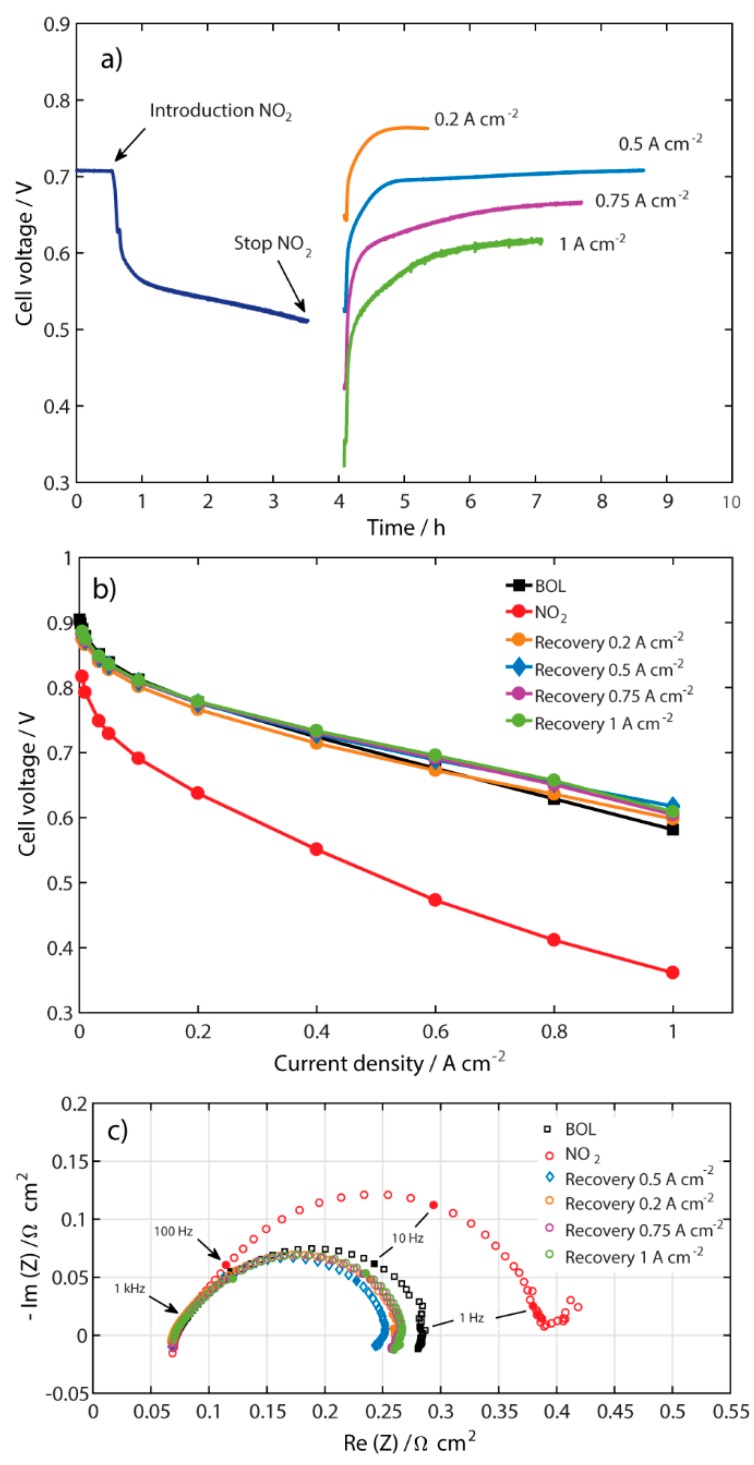
(**a**) Performance with 100 ppm NO_2_ and recovery at current densities 0.2, 0.5, 0.75, and 1 A cm^−2^ and constant 110 mL min^−1^ air flow; (**b**) polarization curves; and (**c**) galvanostatic EIS measurements at 0.5 A cm^−2^ at BOL, after contamination, and after recovery at each current density.

Another strategy investigated was to recover the contaminated MEA at different current densities, as shown in Figure 3a. The same contamination procedure was done as in Figure 1a and the different controlled current densities for recovery process were 0.2, 0.5, 0.75, and 1 A cm^−2^. It can be seen that all of the performance recovery measurements reached a steady state at their respective current densities. As also seen in the experiments with different air flow rates, the voltage increases abruptly after the NO_2_ is switched off and replaced with clean air, which here again may be related to the rapid removal of NO_2_ from the Pt-catalyst. The necessary time to reach steady state after the recovery process was different for the different current densities, and decreased as the current density increased above 0.5 A cm^−2^. Surprisingly, the time to reach steady state at the recovery current density of 0.2 A cm^−2^ was the shortest. This is a sign of a different mechanism that occurs at this specific current density. From our previous study using cyclic voltammetry in inert media with no water production [4], it was seen that around the range of potential that this current density corresponds to (0.65–0.76 V), reduction of NO_3_^−^ to NO_2_^−^ may occur. However, in the present experiments, water is produced at the cathode side and may react with NO_2_ producing HNO_3_ and NO, as shown in Equation (1). It can be pointed out that nitric acid in water is normally present as NO_3_^−^ [13].

3 NO_2_ + H_2_O → 2H^+^ + 2 NO_3_^−^ + NO (1)

The range of potentials in which the performance is recovered at the current density of 0.2 A cm^−2^, i.e. 0.65–0.76 V (Figure 3a), is almost the same as the one in the inert media [4]; therefore, NO_3_^−^ may be reduced to NO_2_^−^ in the present experiments as well. Additionally, NO contamination is similar to CO contamination in that both contaminants affect the catalyst layer and, at low current densities in presence of O_2_, NO is removed from the catalyst. This may explain the faster recovery at lower current densities (0.2 A cm^−2^), while at higher current densities the NO contamination is more severe and oxygen is predominantly producing water through ORR. This suggests that chemical reactions may be present and followed by electrochemical reaction, in the same way as discussed by Chen et al. [10].

Figure 3b shows the respective polarization curves after the recovery process at different current densities. The performance after the recovery process done at the current densities of 0.5, 0.75, and 1 A cm^−2^ overlapped with that at BOL until 0.4 A cm^−2^ in the polarization curve. At higher current densities, they still overlapped each other but they differed from the BOL, in a similar way as in Figure 2b. However, the behavior of the performance after the recovery process done at 0.2 A cm^−2^ was different. It is seen that this performance was not fully recovered; even though it reached a steady state during the recovery process, as shown in Figure 3a, it still had 15 mV left to full recovery. For this recovery current density, a better performance than at BOL was seen at current densities higher than 0.6 A cm^−2^. A possible reason it did not fully recover at the current density of 0.2 A cm^−2^ may be the formation of intermediate species around 0.7 V that may have affected the performance.

Figure 3c shows the EIS spectra after the recovery process at different current densities. As in Figure 2c, there is no significant difference in the HFR and the polarization resistance decreases after the recovery process. The lowest polarization resistance was observed for the recovery at 0.5 A cm^−2^.

### 2.4. Other Types of Recovery

**Figure 4:**
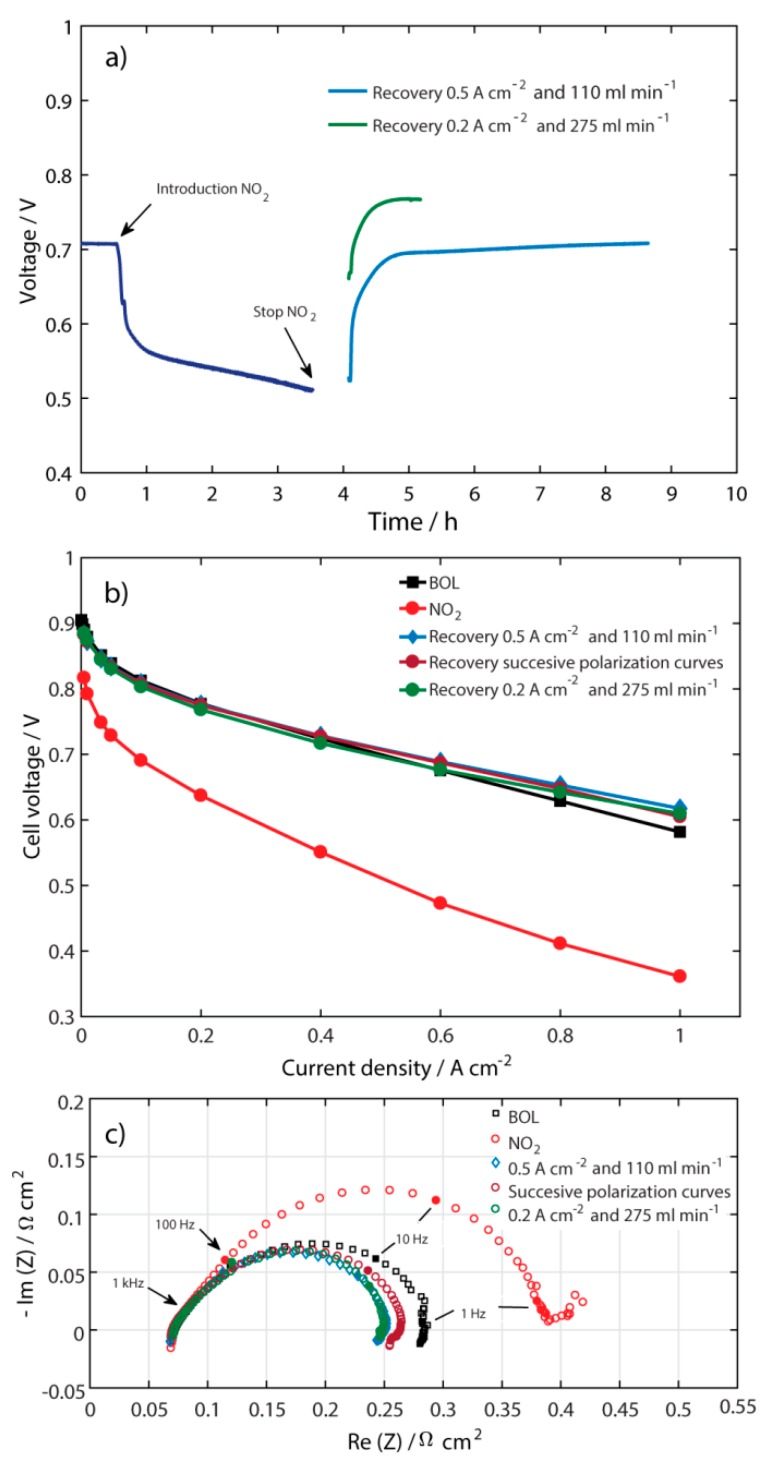
(**a**) Performance with 100 ppm NO_2_, recovery at 0.5 A cm^−2^ and 110 ml min^−1^, and recovery at 0.2 A cm^−2^ and 275 ml min^−1^; (**b**) Polarization curves at BOL, after contamination with NO_2_, after recovery at 0.5 A cm^−2^ and 110 mL min^−1^, after recovery by successive polarization curves with 110 mL min^−1^ as a constant air flow, and after recovery at 0.2 A cm^−2^ and 275 mL min^−1^; and (**c**) EIS spectra for the measurements done in (**b**).

Thus far, it has been shown that the recovery process time after contamination with NO_2_ can be shortened. A summary of times for recovery, from the used recovery methods, is shown in Table 1. The two shortest times were found to be at current density of 0.2 A cm^−2^ and air flow of 275 mL min^−1^. Therefore, these two operating conditions were combined to potentially obtain an even shorter recovery time (Figure 4a). Additionally, successive polarization curves after contamination with NO_2_ were tried as a recovery method. For this experiment, the polarization curves were conducted in galvanodynamic mode at a step rate of 5 mA s^−1^. Figure 4b shows the polarization curves for the latter two recovery methods compared with the polarization curves at 0.5 A cm^−2^ and 110 mL min^−1^, at BOL, and directly after contamination with 100 ppm NO_2_. The recovery time for the 0.2 A cm^−2^ and 275 mL min^−1^ air flow was 66 min, i.e. the time was reduced by 10 min compared with the recovery process at 0.2 A cm^−2^ and 110 mL min^−1^. This indicates that the airflow rate is an important parameter; it seems that NO_2_ can be removed from the Pt-catalyst by the air, and/or that O_2_ is participating in chemical and electrochemical reactions in the removal of NO_2_ species, as mentioned in the Section 2.3. The recovery time when performing successive polarization curves was 35 min, which was found to be the fastest way to recover the performance of the fuel cell contaminated with NO_2_.

It is worth mentioning that none of the polarization curves after contamination with 100 ppm NO_2_ reached values around 0.2 V. The lowest potential (0.35 V) was reached at a current density of 1 A cm^−2^. Therefore, the reduction of NO_2_^-^ to N_2_O and/or NH_2_OH [4] may not be present in these set of experiments.

A theoretical prediction for the recovery of NO_2_ was made by St-Pierre et al. [12]; however, in their prediction, they did not include all processes in the fuel cell that may be affected by degradation, such as ohmic losses and mass transport, which explains the results obtained. It would be interesting to investigate performance recovery in a wider current density range. 

The EIS measurements in Figure 4c show that, even though the shortest recovery time was reached by the successive polarization curves, the spectra of the experiment at 0.5 A cm^−2^ and 110 mL min^−1^ together with the spectra at 0.2 A cm^−2^ and 275 mL min^−1^ were those that had the lowest polarization resistance.

### 2.5. Comparison of Two Operation Techniques

**Figure 5:**
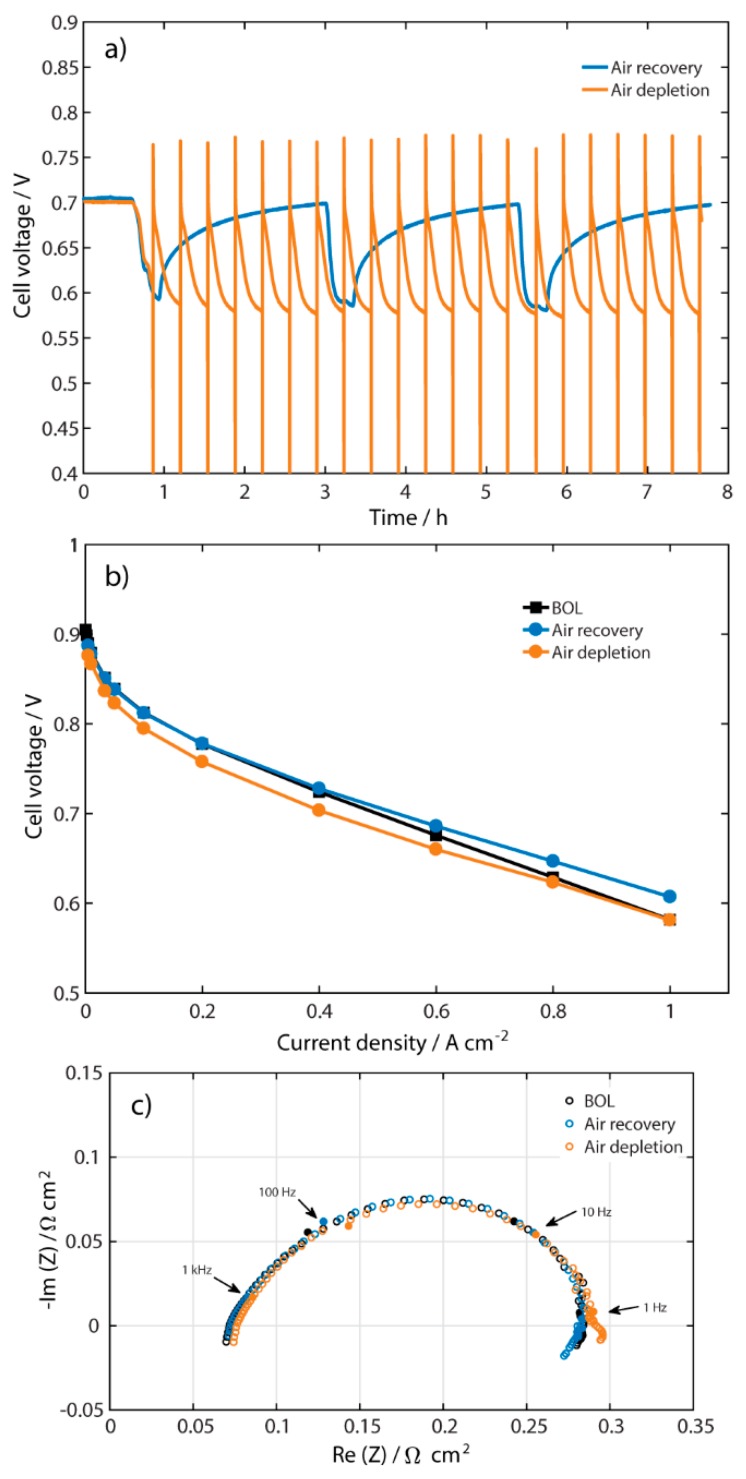
(**a**) Transient cell voltage when introducing 50 ppm NO_2_ to the cathode air flow at 0.5 A cm^−2^ for two different strategies, namely air recovery (blue) and air depletion (orange); (**b**) polarization curves; and (**c**) galvanostatic EIS measurement at 0.5 A cm^−2^ at BOL and after testing the two strategies.

Finally, two operation techniques for the cathode were applied and compared by introducing 50 ppm of NO_2_ in different ways, as shown in Figure 5a, with the goal to suggest online application in a fuel cell car. This concentration was chosen because it is more probable to find 50 ppm NO_2_ in air than 100 ppm or higher concentrations. In both experiments, the cell was first stable for 30 min, keeping the same potential. The experiment done with air recovery (blue line) consisted of introducing NO_2_ with balance of air to the cathode for 20 min, and then recovering the performance with clean air for 2 h. The same sequence was repeated three times. In the experiment with air depletion (orange line), the NO_2_ contaminated air was fed to the cathode during 20 min, after which the air gas flow was switched off until the potential reached 0.01 V. At that point, the gas was switched on again. This experiment was made with the purpose to sweep the cell voltage within a wide range in order to let the fuel cell to recover quickly. The procedure was repeated 21 times to be comparable in time with the air recovery technique. Figure 5a shows a complete reversibility during the air recovery technique, in which all cycles reached the initial value (0.7 V). In both techniques, a lower cell voltage is seen after the 20 min with NO_2_ compared to the first contamination cycle, but no significant difference is shown between the cycles. The outcome of the air recovery is in accordance with the results of Mohtadi et al. [9], who obtained a complete recovery after three cycles with 5 ppm of NO_2_. On the other hand, the cell performance obtained by Yang et al. [7] did not reach the initial value after recovery. However, they used a different pressure (0.5 bar), and it is known that the pressure is an important parameter concerning recovery of a fuel cell contaminated by NO_2_ [6].

At the end of each experiment, a polarization curve was recorded (see Figure 5b). The figure shows that the strategy with air depletion resulted in a lower performance after 7 h of operation, which might be caused by deterioration of the electrode due to peroxide formation at low electrode potentials [14,15,16]. On the other, the polarization curve after air recovery revealed a complete recovery of the Pt-catalyst, and even better performance at current densities higher than 0.4 A cm^−2^. The air recovery technique suggests that NO_2_ is only attached to the Pt-catalyst of the electrode and that it can be easily removed by air, apparently, without affecting other components.

EIS was also conducted at the end of each experiment (Figure 5c). The figure shows no significant difference between the two strategies, although the HFR of the air depletion spectrum increased only corresponding to about 3 mV when compared to beginning of life, but this is in the range of error.

These results show that it is possible to operate a specific technique online in a fuel cell vehicle in order to deal with NO_2_ air pollution. However, the technique must be adapted to a more realistic drive cycle. Operating parameters such as air flow rate and current density can also possibly be incorporated in a recovery method to keep good performance after NO_2_ contamination.

## 3. Materials and Methods 

The experimental set up used in this investigation was the same as used in our previous study with NO_2_ [4]. A commercial fuel cell hardware from Fuel Cell Technologies, Inc., and a commercial membrane electrode assembly (MEA) (Gore^™^Primea^®^ 5641), with catalyst loadings of 0.45 mg cm^−2^ Pt-alloy on the anode and 0.4 mg cm^-2^ Pt on the cathode, were used in all of the experiments. The same type of gas diffusion layer (GDL) (Carbel^™^) was used at both anode and cathode. The geometric electrode area used was 1.5 cm^2^. The cell temperature was kept at 80 °C and 1 atm, and the humidification of the gases was 90% RH. The gas cylinder used was the same as in [4], and the contamination flow was controlled by an Alicat Scientific mass flowmeter.

The electrochemical characterization procedure was the same as in our previous study [4]. For the contamination step, a galvanostatic measurement was done, followed by polarization curve measurement and electrochemical impedance spectroscopy (EIS) by use of a Solartron Interface SI1287 potentiostat together with a 1255 frequency response analyzer, controlled by CorrWare software. For the EIS, an AC amplitude of 60 mA (roughly corresponding to 3–15 mV depending on frequency and operating conditions) was used in the frequency range between 10 kHz and 30 mHz. It was assumed, in all experiments, that the electrical bulk and contact resistances were not affected by the introduction of NO_2_, and that the high frequency resistance is related to the resistance of the membrane. 

## 4. Conclusions

The results show that it is possible to find adequate performance recovery methods that can be applied in a fuel cell car in a real traffic situation where large amounts of NO_2_ are present. In the experiments done in galvanostatic mode at 0.5 A cm^−2^ with air flow of 110 mL min^−1^, a significant potential drop was observed due to the presence of NO_2_ in the cathode air. This performance loss was however totally recovered after 4.5 h with clean air. The study shows that it is possible to significantly decrease the time for performance recovery by running successive polarization curves or by applying 0.2 A cm^−2^ and an air flow of 275 mL min^−1^. Two operation techniques that can be used online in a fuel cell vehicle were also tested: air recovery and air depletion. The air recovery technique was found to be the best option for recovery of performance. Therefore, we assume that air can pull out the NO_2_ molecules that surround the Pt-catalyst to free up the active site at higher current densities; however, at the current density of 0.2 A cm^−2^, possibly a different contamination mechanism occurs.

## Figures and Tables

**Table 1:** Summary of the performance recovery time after introduction of 100 ppm NO_2_ to the cathode air flow. Pure H_2_ was used at the anode.

Recovery at Different Air Flows Rates at 0.5 A cm^−2^		Recovery at Different Current Densities at 110 mL min^−1^	
mL min^−1^	min	A cm^−2^	min
110	274	0.2	76
165	213	0.5	274
220	161	0.75	217
275	106	1	181
